# An Asymptomatic 42-Year-Old Woman With Randomly Distributed Semicalcified Pulmonary Nodules

**DOI:** 10.1016/j.chpulm.2026.100237

**Published:** 2026-03-10

**Authors:** Ilias E. Dimeas, Vasilios Tzilas, Konstantinos Tourlakopoulos, Periklis G. Foukas, Cormac McCarthy, Zoe Daniil

**Affiliations:** a School of Medicine, University College Dublin, Dublin, Ireland; b Department of Respiratory Medicine, St. Vincent’s University Hospital, Dublin, Ireland; c Department of Respiratory Medicine, Faculty of Medicine, University of Thessaly, Biopolis, Larissa, Greece; d 2nd Pulmonary Medicine Department, General University Hospital “Attikon,” Athens, Rimini, Athens Medical School, National and Kapodistrian University of Athens, Athens, Greece; e 2nd Department of Pathology, National and Kapodistrian University of Athens, School of Medicine, Attikon University Hospital, Athens, Greece; f School of Medicine, University College Dublin, Dublin, Ireland

## Abstract

A 42-year-old woman with a 25 pack-year smoking history, who quit 5 years prior to presentation, was referred to our outpatient clinic for reevaluation of multiple bilateral pulmonary nodules first identified 4 years earlier on chest computed tomography, performed following a self-limited flu-like illness with transient rash and nonproductive cough. Her symptoms resolved without intervention, and she remained clinically stable in the years that followed, with only occasional episodes of dry cough; however, the radiologic findings and her medical history prompted progressive diagnostic workup.

Her medical history included non-Hodgkin lymphoma treated with chemotherapy 9 years before referral, with a documented relapse 5 years earlier and no evidence of active disease in the preceding 4 years. She had undergone total thyroidectomy for benign thyroid nodules 8 years before referral and partial nephrectomy for clear cell renal carcinoma 3 years earlier. At the time of referral, she was receiving thyroid hormone replacement therapy and a long-acting beta-agonist/long-acting muscarinic antagonist inhaler prescribed by her general practitioner for intermittent cough. She had no known TB exposure, no significant travel history, and no environmental or domestic risk factors, but reported occupational exposure from prior work in a charcoal grill restaurant. She had no history of aspiration episodes and reported no symptoms suggestive of chronic aspiration or dysphagia.

Persistent abnormalities on serial imaging prompted extensive investigation over the years, including 2 surgical biopsies and multiple diagnostic studies, none of which yielded a definitive diagnosis. Despite the persistent radiographic findings, including nodules of varying size and semicalcified appearance, her clinical status remained stable, and no treatment had been initiated. She was ultimately referred to the department of respiratory medicine and admitted for further evaluation.

## Physical Examination Findings

On physical examination at the time of referral, the patient appeared well and in no acute distress. Vital signs, including oxygen saturation, were within normal limits. Pulmonary auscultation revealed normal breath sounds without crackles, wheezing, or rubs. Cardiovascular and abdominal examinations were unremarkable. No digital clubbing, lymphadenopathy, rash, or other cutaneous abnormalities were observed. Minor bilateral thoracic surgical scars were noted, consistent with prior lung biopsies. No additional abnormalities were identified on comprehensive physical examination.

## Diagnostic Studies

At the time of admission, a comprehensive diagnostic workup was initiated, acknowledging the extensive, though inconclusive, investigations already performed over the preceding 4 years. Given the persistence of radiographic abnormalities in a clinically stable, asymptomatic patient with complex oncologic history, our approach was to both validate prior results and broaden the differential by including underrecognized entities.

**Figure 1:**
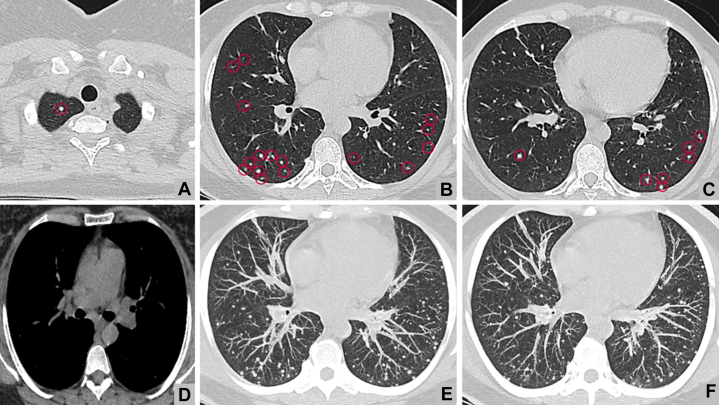
A-F, Initial chest CT scan showing innumerable, randomly distributed bilateral semicalcified pulmonary nodules. A-C, Nodules in various lung regions are demonstrated. D, Borderline mediastinal lymphadenopathy is shown. E-F, Maximum intensity projection reconstructions emphasizing the diffuse and extensive distribution of pulmonary nodules. Selected nodules are marked with crimson circles for clarity.

**Figure 2:**
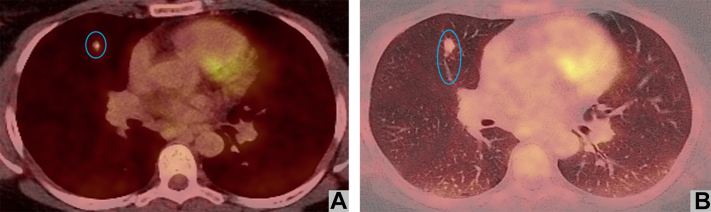
A-B, PET-CT scan evaluation of the right upper lobe nodule. A, Increased metabolic activity (maximum standardized uptake value, 6.8) in the nodule on PET imaging. B, Corresponding fused PET-CT image, localizing the uptake to the right upper lobe lesion. Scattered low-grade parenchymal activity was observed; however, no hypermetabolic activity was detected in other nodules, lymph nodes, or distant sites. The nodule is marked with a blue circle in both panels.

The initial chest CT scan (Fig 1) revealed innumerable, randomly distributed bilateral semicalcified pulmonary nodules with borderline mediastinal lymphadenopathy. Two years later, a follow-up CT scan demonstrated a modest increase in size of a nodule in the right upper lobe, without changes regarding the mediastinal lymphadenopathy. This prompted a PET-CT scan (Fig 2), which showed a maximum standardized uptake value of 6.8 in that lesion, without evidence of hypermetabolic activity in other nodules, lymph nodes, or distant sites. Notably, during this 2-year interval, the patient was also diagnosed with clear cell renal carcinoma and underwent partial nephrectomy, contributing to the delayed reassessment.

Bronchoscopy with endobronchial ultrasound transbronchial needle aspiration and bronchoalveolar lavage (BAL) was performed. Endobronchial ultrasound transbronchial needle aspiration revealed reactive lymph node tissue without evidence of malignancy or granulomatous inflammation. BAL cytology was negative for malignancy, including recurrence of non-Hodgkin lymphoma or metastatic disease. Flow cytometry of BAL fluid showed no clonal or atypical populations, with a CD4/CD8 ratio of 0.3 and otherwise normal immune cell content. Microbiological studies, including gram stain, cultures, acid-fast bacilli stain and culture, fungal culture, and broad-range polymerase chain reaction (PCR) assays for mycobacteria and fungi, were negative. Serum and BAL β-D-glucan and galactomannan assays, and serologies for endemic mycoses, were unremarkable. Mantoux testing was 0 mm, and interferon-gamma release assay was negative. Serum autoimmune and inflammatory markers, including antinuclear antibodies, antineutrophil cytoplasmic antibodies, serum angiotensin-converting enzyme, immunoglobulin levels (including IgG subclasses), serum amyloid A, rheumatoid factor, and anticitrullinated peptide antibodies, were all within normal limits. Full blood count was normal without eosinophilia. HIV serology was negative, and whole-blood lymphocyte immunophenotyping did not reveal evidence of immunodeficiency. Systemic evaluations by ear, nose, and throat, neurology, nephrology, and echocardiography for vegetations, mitral valve stenosis, or any regurgitation of heart valve were unremarkable. Radiologic review found no fat-density lesions on CT scan to suggest cholesterol granulomas or lipoid pneumonia.

Because of the inconclusive results and radiographic concern for neoplasia, a surgical wedge resection of the right upper lobe was performed shortly thereafter. Histopathology revealed necrotizing granulomatous inflammation without malignancy, vasculitis, or identifiable organisms. Areas of central necrosis were surrounded by fibrosis, histiocytes, and multinucleated giant cells. Histologic Congo red staining, CD1a, and IgG4 immunostaining on biopsy tissue were negative. The findings were histologically compatible with necrotizing sarcoid granulomatosis; however, this diagnosis was considered unlikely given the absence of compatible clinical, radiologic, or laboratory features. No extrapulmonary organ involvement suggestive of sarcoidosis was identified, and other histologic mimics including vasculitis, Langerhans cell histiocytosis, pneumoconiosis, organizing pneumonia, and Erdheim-Chester disease were excluded based on immunohistochemistry, histologic, and clinical review.

**Figure 3:**
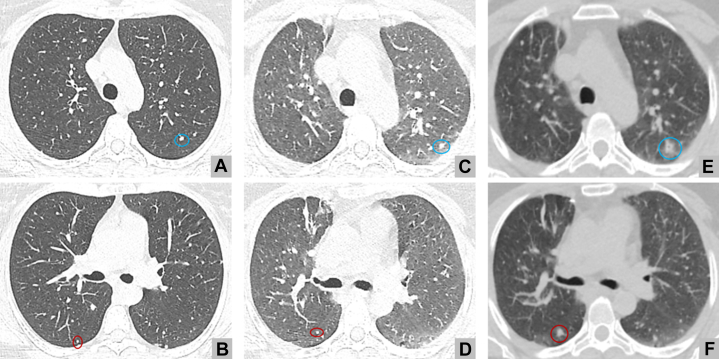
A-F, Serial chest CT imaging demonstrating interval growth of pulmonary nodules. A-B, Initial CT scan (corresponding to Fig 1). C-D, Follow-up scan 2 y later. E-F, Most recent CT scan, approximately 1 y after the previous imaging. The left upper lobe nodule, marked in a blue circle, and the right lower lobe nodule, marked in a crimson circle, both demonstrate interval growth. Notably, the nodule in the left upper lobe (panel E) exhibits central lucency, suggestive of necrosis.

**Figure 4:**
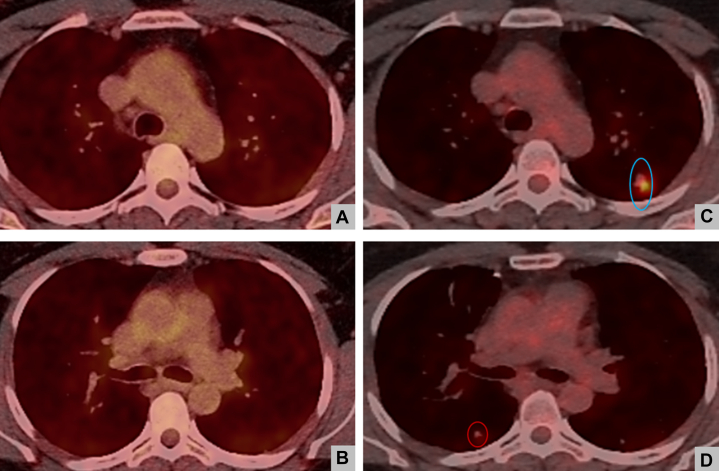
A-D, PET-CT scan comparison demonstrating new metabolic activity in pulmonary nodules. A-B, PET-CT scan from 1 y earlier (corresponding to Fig 2), without evidence of the metabolically active nodules now seen in panels C and D. C-D, Increased uptake in 2 nodules: the left upper lobe (maximum standardized uptake value [SUVmax], 7.0; blue circle) and the right lower lobe (SUVmax 4.6, crimson circle). Previously noted scattered low-grade parenchymal activity has decreased.

Because the patient was asymptomatic and there was still no definitive diagnosis after a wedge biopsy, the managing team opted for close clinical and radiologic surveillance. Approximately 1 year later, repeat CT imaging (Fig 3) demonstrated interval growth of new nodular lesions in the left upper and right lower lobes, whereas some preexisting nodules had regressed. PET-CT (Fig 4) demonstrated increased uptake, with a maximum standardized uptake value of 7.0 in the left upper lobe and 4.6 in the right lower lobe. A second bronchoscopy with BAL showed normal findings, including normal cytology, and flow cytometry again showed no aberrant populations. A second surgical biopsy via video-assisted thoracoscopic surgery was performed. Histology once more demonstrated necrotizing granulomas with surrounding histiocytes, lymphocytes, foreign body-type giant cells, and prominent calcium salt deposition, consistent with semicalcified necrotic nodules. No organisms or malignant cells were identified, and all microbiology tests were negative as before. Despite thorough and repeated evaluation, no definitive etiology was established, and the patient remained under observation.

After her referral to our team, a new chest CT scan demonstrated stable yet heterogeneous bilateral pulmonary nodules, with some lesions slightly increased and others decreased in size. The patient also reported that she had permanently ceased occupational exposure related to her prior work in a charcoal grill restaurant. Given her understandable reluctance to undergo a third invasive biopsy, a noninvasive reassessment was prioritized. Bronchoscopy revealed normal mucosa and no endobronchial abnormalities. BAL cultures and molecular studies were negative for bacterial, mycobacterial, and fungal pathogens; cytology was negative for malignancy, and flow cytometry again demonstrated no clonal or atypical populations. Endobronchial ultrasound did not identify lymph nodes with size or morphology meeting criteria for sampling. A repeat immunologic evaluation confirmed intact immune function, without evidence of acquired immunodeficiency or autoimmune disease.

**Figure 5:**
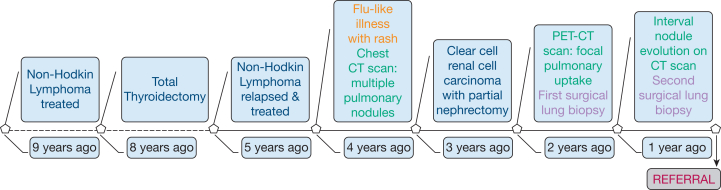
Timeline summarizing major clinical and radiologic milestones. Shown are the diagnoses and management of non-Hodgkin lymphoma and clear cell renal cell carcinoma, the intervening flu-like illness with rash, and the evolution of pulmonary nodules on imaging. The figure highlights the temporal relationship between periods of clinical stability, surgical interventions, and radiologic changes.

A consolidated timeline summarizing key clinical and radiologic events is shown in Figure 5. Given the persistence of calcified necrotizing nodules in an immunologically reconstituted patient with prior oncologic history, and after the exclusion of more common infectious, neoplastic, inflammatory, and environmental etiologies, a viral-specific PCR was considered and ordered on formalin-fixed paraffin-embedded tissue from the most recent surgical biopsy.

*What is the diagnosis?*

*Diagnosis:* A positive varicella-zoster virus PCR from necrotizing granulomatous lung tissue, in the context of randomly distributed, persistent semicalcified pulmonary nodules in an immunologically reconstituted, clinically stable adult with a prior flu-like illness and rash, establishes the diagnosis of healed varicella-zoster virus pulmonary infection.

## Discussion

This case highlights the diagnostic complexity and teaching value of persistent, randomly distributed, calcified pulmonary nodules in a clinically stable patient, ultimately attributed to a healed varicella-zoster virus (VZV) infection. The convergence of oncologic history, immune reconstitution, and atypical radiologic progression made this an especially challenging and instructive diagnostic journey. The differential diagnosis for diffuse, bilateral, randomly distributed, semicalcified pulmonary nodules is broad. In this patient, the initial concern centered on recurrent or metastatic malignancy, particularly given her history of non-Hodgkin lymphoma and clear cell renal carcinoma. Other important differentials included infectious etiologies such as miliary TB, endemic fungal infections, and parasitic disease; immune-mediated conditions such as sarcoidosis, vasculitis, and necrotizing sarcoid granulomatosis; pneumoconioses related to charcoal exposure; and rare entities such as Langerhans cell histiocytosis and Erdheim-Chester disease. Repeated episodes of aspiration were also considered in the differential; however, no clinical supported this etiology. Comprehensive histopathology, imaging, microbiology, and immunologic testing enabled systematic exclusion of these differential diagnoses.

Acute VZV pneumonia typically occurs in immunocompromised adults with diffuse nodular infiltrates, ground-glass opacities, and consolidation. In immunocompetent individuals, it is rare and usually self-limited; however, complications such as myelitis and nodular lung sequelae have been described. Healed VZV pneumonia can manifest as randomly distributed, calcified pulmonary nodules, frequently misinterpreted as metastatic disease. Importantly, symptoms may be mild or absent, and the initial viral phase often goes unrecognized or unreported unless retrospectively elicited, as in this case. Given the broad clinical spectrum of VZV-related lung disease, in the absence of active systemic symptoms, the detection of VZV genetic material in formalin-fixed lung tissue by PCR serves as the gold standard for diagnosing healed pulmonary infection. This molecular confirmation must be interpreted alongside histopathologic features such as necrotizing granulomas with multinucleated giant cells and fibrosis, and a radiographic pattern of randomly scattered, nonprogressive nodules. The presence of prior rash or flu-like illness, even if retrospectively recalled, increases diagnostic plausibility.

This case also provides insight into the postinfectious immune dynamics of healed VZV. Although the mechanisms remain speculative, the observed pattern, initial lesion shrinkage followed by paradoxical regrowth despite immune recovery, is consistent with hypotheses proposed in prior literature. During immunosuppression, whether due to chemotherapy or the malignancy itself, reduced cellular immunity may have allowed viral persistence but suppressed granulomatous inflammation. On immune reconstitution, the restoration of immune cell activity may have promoted exaggerated granulation and fibrotic repair, leading to radiologic progression rather than true viral reactivation. This is supported by the absence of active viremia or systemic signs on repeat serologies and imaging. This case reinforces the importance of molecular diagnostics in atypical granulomatous lung disease and the nuanced interpretation of radiologic changes in immune-reconstituted hosts. In patients with compatible history, imaging, and histology, healed varicella pneumonia should be considered, especially in cases where alternative etiologies have been rigorously excluded.

### Clinical Course

The most striking aspect of this case is that a clinically silent and ultimately benign process warranted 2 justified lung biopsies in a patient with a complex oncologic background, due to persistent, metabolically active pulmonary nodules concerning for malignancy. Throughout the multiyear course, the nodules exhibited dynamic radiologic behavior, with some increasing and others decreasing in size, which further complicated the diagnostic picture. Importantly, the patient was immunologically reconstituted at the time of referral, raising the possibility that immune recovery itself contributed to the evolving radiographic changes. The absence of symptoms and systemic findings further supports this interpretation. After the tissue diagnosis of healed VZV pneumonia was established, additional investigations were performed. VZV PCR testing of blood and BAL fluid were negative. Serology showed positive VZV IgG, negative IgA, and low-level IgM below diagnostic thresholds; repeat titers after 4 weeks were unchanged. Given the lack of viremia or active replication, and after multidisciplinary consultation with infectious diseases, hematology, and immunology, antiviral therapy was not initiated. The patient remained asymptomatic, and no further invasive testing was deemed necessary. Annual low-dose CT scan was recommended for surveillance. A follow-up low-dose chest CT scan performed 1 year later showed stable findings. From a psychosocial standpoint, the prolonged diagnostic uncertainty had been a source of anxiety, and the patient ultimately expressed significant relief on receiving a definitive and benign diagnosis.

## Clinical Pearls

1. *Healed VZV pneumonia may be incidentally identified years after infection as persistent, randomly distributed, calcified pulmonary nodules, often radiologically mistaken for metastatic disease.*
2. *Tissue PCR for VZV DNA remains the gold standard for confirming healed VZV pulmonary infection, particularly in asymptomatic patients with necrotizing granulomas and compatible clinical and radiologic findings.*
3. *Immune reconstitution after chemotherapy or malignancy may trigger delayed granulomatous inflammation, leading to nodule progression without necessarily indicating active infection.*
4. *Healed VZV infection can be entirely asymptomatic; the absence of systemic symptoms does not exclude prior viral pneumonitis.*
5. *The differential diagnosis of pulmonary granulomas should include viral infections such as VZV, which are often overlooked in standard references.*

## Financial/Nonfinancial Disclosures

None declared.
